# Sustainable Strategies for Full Use of *Miscanthus*: Biodegradable Seedling Pots and Lignin-Based Fertilizers

**DOI:** 10.3390/polym18101181

**Published:** 2026-05-12

**Authors:** Jiyu Guan, Chao Liu, Guang Yu, Mohammad Ali Asadollahi, Chunxiang Fu, Wangda Qu, Bin Li

**Affiliations:** 1 College of Life Sciences, Qingdao Agricultural University, Qingdao 266109, China; 2 Qingdao New Energy Shandong Laboratory, System Integration Engineering Center, Qingdao Institute of Bioenergy and Bioprocess Technology, Chinese Academy of Sciences, Qingdao 266101, China; 3 Department of Biotechnology, Faculty of Biological Science and Technology, University of Isfahan, Isfahan 81746-73441, Iran

**Keywords:** *Miscanthus*, pretreatment, wet foaming, lignin, fertilizer, biodegradable seedling pots

## Abstract

*Miscanthus* (*Panicum virgatum* L.), a biomass material known for its rapid growth and high biomass yield, is considered a suitable resource for producing biobased materials. Nevertheless, the dense and complex structure of *Miscanthus* hinders its full utilization. In this study, alkaline sulfite pretreatment of *Miscanthus* was carried out to separate the cellulosic fiber fraction and sulfonated lignin. Then, the fiber fraction was used to prepare biobased seedling pots via the wet foaming technique, and the maximum compressive strength of the prepared seeding pot could reach 1317 kPa. The surface coating of the seeding pot with wood wax oil further improved its hydrophobicity and water resistance. Furthermore, the resulting seedling pot with good biodegradability can be used to replace the petroleum-based plastic seedling pot, which could reduce plastic pollution. In addition, the fractionated sulfonated lignin was directly utilized as a fertilizer, showcasing a 6% increase in root and stem height of cabbage and a 15% rise in biomass (dry weight), compared to the humic acid treatment group. Therefore, this work offers a promising and sustainable strategy for the comprehensive utilization of *Miscanthus*, which can also be a beneficial reference for the better use of other kinds of lignocellulosic biomass.

## 1. Introduction

As fossil fuels are progressively depleted, energy shortages and environmental pollution have become increasingly urgent challenges, and the search for effective alternatives has therefore intensified. Lignocellulosic biomass represents the only renewable carbon resource available on a large scale on the Earth, indicating that its utilizations as a substitute for conventional fossil-based sources hold considerable potential. *Miscanthus* is widely considered one of the most promising nonwoody-fiber energy crops owing to its high biomass productivity and favorable energy properties (i.e., high content of carbohydrates) [1]. Native to East Asia, *Miscanthus* is a perennial grass capable of producing 10–40 t dry matter per ha annually within just two to three years after planting [2,3]. *Miscanthus* exhibits rapid growth, high adaptability efficiency, and efficient utilization of nitrogen, water, and light, alongside strong adaptability to diverse climatic conditions, including tolerance to low winter temperatures [4]. Also, *Miscanthus* contributes to ecological restoration: its cultivation can reduce soil erosion, enhance nutrient cycling, and improve soil texture, structure, and water-holding capacity, as well as adsorb heavy metals in contaminated soils [5]. Furthermore, planting *Miscanthus* on marginal lands, such as saline-alkali or sandy soils, can generate substantial biomass without competing with food crops for arable land. Consequently, the large-scale cultivation of *Miscanthus* not only promotes efficient land use but also enhances carbon sequestration, highlighting its broad environmental and economic value.

Current utilization pathways for *Miscanthus* can be categorized into two major categories: energy utilization and material utilization. Energy utilization primarily involves converting *Miscanthus* into advanced biofuels (e.g., bioethanol, biobutanol, sustainable aviation fuel) [6,7]. For instance, Han et al. [8] pretreated *Miscanthus* with dilute NaOH, and the resulting ethanol concentration reached 59.2 g/L after the following enzymatic saccharification and fermentation. In addition to biofuel production, *Miscanthus* can also serve as a raw material for producing various biomaterial products (such as paper-based products [9], Lyocell fibers [10], and nanocellulose) due to its high content of cellulose and high quality of cellulosic fibers. For instance, Barbash et al. [11] applied a two-step pretreatment method of peracetic acid treatment and alkaline treatment to remove lignin and part of hemicellulose from *Miscanthus*. Subsequently, ultrasonic treatment was adopted for the cellulosic fraction to obtain nanocellulose with a crystallinity index of 76.5%, which was further used to prepare cellulose nanopaper with a transparency of 82% and a tensile strength of 195 MPa. However, the use of lignin was not reported in their work. Lignin contains many active groups, such as phenolic hydroxyl and carboxyl groups. Other functional groups, like sulfonic groups and amino groups, can also be introduced into lignin molecules [12]. These active groups could chelate metal ions essential for plant growth [13]. Notably, sulfonated lignin (SL, prepared by sulfonation of lignin [14], or separated via sulfite pretreatment of lignocellulose [15]) could be degraded by soil microorganisms into humic acid (HA), thereby increasing the organic matter content in the soil [16], promoting plant growth, and serving as a fertilizer. Consequently, to mitigate resource waste and enhance resource value, it is necessary to develop a novel comprehensive utilization pathway for the components of *Miscanthus*.

*Miscanthus* contains a complex lignocellulosic structure, and the three primary constituents of cellulose, hemicellulose, and lignin were tightly combined together to create a strong natural recalcitrance, hindering the full exploitation of these components. Pretreatment is able to disrupt this biomass barrier, facilitating the efficient separation of its constituents, and thus, pretreatment is a critical step in enhancing their utilization. Based on reaction mechanisms, pretreatment strategies are typically grouped into physical (e.g., ball milling, disc refining, and extrusion) [17], biological (e.g., use of lignin-degrading enzymes) [18], and chemical methods. Chemical pretreatment, using dilute acids, alkalis, or organic solvents, operates at controlled temperatures and pressures to cleave chemical bonds among lignocellulosic components and disrupts biomass structure by efficiently removing hemicellulose or lignin, thus reducing recalcitrance and enhancing cellulose accessibility or processibility [19]. Among chemical methods, sulfite pretreatment with reagents of sodium sulfite or ammonium sulfite can introduce sulfonic groups onto lignin macromolecules, thus increasing hydrophilicity, solubilization, and fractionation of lignin from lignocellulose. Also, sulfite pretreatment conditions are generally milder compared to other chemical methods (e.g., dilute acid or organic solvents), which can reduce degradation of cellulose and hemicellulose during delignification [20]. Moreover, the fractionated SL is an amphiphilic polymer with considerable economic value, owing to its applications (e.g., dispersant [21], concrete water reducer [22], adhesive [23], fertilizer [24]) across multiple industries.

**Figure 1:**
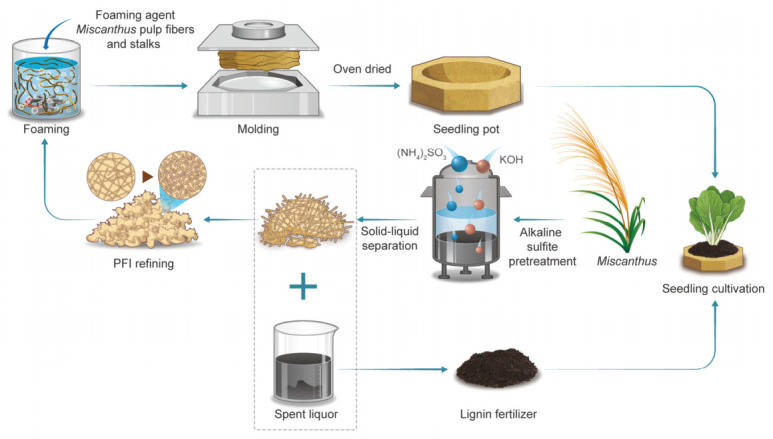
Flowchart of the full utilization of *Miscanthus*.

During the cultivation of plants (e.g., vegetables, flowers, *Miscanthus*), seedling pots are widely used or highly recommended for use. Yet, currently, the market is dominated by plastic seedling pots, which are non-biodegradable and often end up in landfills or incinerators, releasing harmful pollutants into the environment [25,26]. Recently, it has been reported that cellulose pulp fibers can be foamed (via wet foaming) to produce a relatively new type of lightweight and biodegradable bio-foam with good water stability [27]. The wet foaming process of cellulosic fibers mainly includes surfactant foaming, mold shaping, filtration, and air or oven drying [28], which is a low-cost and eco-friendly technology for preparing cellulosic fiber foam materials [29]. Inspired by this, to better utilize *Miscanthus*, this research proposed a novel comprehensive utilization strategy. Firstly, alkaline sulfite pretreatment was carried out to separate the cellulosic fiber components and lignin. Then, the separated fibers were softened by post-mechanical refining and further used to prepare a biodegradable seedling pot through the wet foaming method at room temperature and atmospheric pressure. The applicability of the prepared seedling pot was comprehensively evaluated. Furthermore, the separated SL served as a potential fertilizer for cabbage cultivation, which was evaluated as well. The whole process for full utilization of *Miscanthus* is schematically shown in Figure 1. This strategy not only offers an innovative technical pathway for the better use of *Miscanthus* resources, but also holds potential significance in mitigating environmental pollution and resource waste.

## 2. Materials and Methods

### 2.1. Materials

*Miscanthus* (*Panicum virgatum* L., particle size of 5–10 mesh) was sourced from Qingdao, Shandong Province, China. The contents of cellulose, hemicellulose, lignin, and extractives of *Miscanthus* were measured to be 36.37 ± 0.41%, 16.28 ± 0.16%, 18.72 ± 0.15%, and 21.75 ± 0.37%, respectively, and the remaining content (approximately 7%) was ash. KOH (assay ≥ 85.0%), NaOH (assay ≥ 96.0%), HCl (36.0%), H_2_SO_4_ (assay ≥ 95.0%), chlorine-type anion exchange resin (717), and sodium-type cation exchange resin (723) were purchased from Sinopharm Chemical Reagent Co., Shanghai, China. (NH_4_)_2_SO_3_ (90 wt.%) was supplied by Shandong Keyuan Biochemical Co., Ltd. (Yantai, China). Alkyl polyglycoside non-ionic surfactant (solid content of 30 wt.%) and wood wax oil were obtained from Shandong Yousuo Chemical Technology Co., Ltd. (Linyi, China) and Shanghai Yuhuan Chemical Co., Ltd. (Shanghai, China), respectively. Polypropylene (PP) seedling pots for comparison and Creamy vegetable seeds (the growth period lasts for 20 to 45 days) were procured from Jinan Huayi Tiantang Flower & Horticulture Co., Ltd. (Jinan, China) and Anhui Jiuqi Seedling Technology Co., Ltd. (Hefei, China), respectively. All chemicals used in this work are of analytical grade and used directly without further purification.

### 2.2. Alkaline Sulfite Pretreatment of Miscanthus

A single-factor experimental design was employed to study the alkaline sulfite pretreatment of *Miscanthus* in a rotary cooking reactor system (VRD-42SD-A, IMT, China National Pulp and Paper Research Institute Co., Ltd., Beijing, China) equipped with four small cooking tubes on the side, and the volume of each tube was 1 L. Specifically, 20 g of dried *Miscanthus* and certain amounts of KOH, (NH_4_)_2_SO_3_, and water were loaded into a small cooking tube, and then the tube was loaded into the cooking reactor for pretreatment. The effects of the following parameters on pretreatment effectiveness were investigated: KOH dosage (0%, 3%, 5%, 7% to the dried *Miscanthus*), (NH_4_)_2_SO_3_ dosage (15% 20%, 25%, 30% to the dried *Miscanthus*), reaction temperature (120 °C, 130 °C, 140 °C), reaction time (1 h, 1.5 h, 2 h) and solid–liquid ratio (1:6, 1:8, 1:10, 1:12). These conditions were evaluated based on their impact on the composition changes of the pretreated biomass, as well as the degree of sulfonation (*DS*) of lignin and lignin removal. After pretreatment, the cooking tubes were immediately cooled down to 40–50 °C with cold tap water. Then, solid–liquid separation was carried out through squeezing with a 300-mesh washing bag. The separated fiber solids were washed with deionized water to neutral pH, and the washed fibers were used to prepare the biodegradable seedling pots. The separated liquid part containing SL and the elements of N and K was directly utilized as fertilizer.

### 2.3. Preparation of Seedling Pots

To investigate the effect of lignin content and post-mechanical refining on the properties of the prepared seedling pots, *Miscanthus* pretreated with 15% or 20% (NH_4_)_2_SO_3_ was subjected to post-mechanical refining using a laboratory-scale PFI refining equipment (PL11-00, TAIST, Xianyang TEST Equipment Co., Ltd., Xianyang, China). During PFI refining, the solid content of fibers was 10 wt.%, and the revolution number of PFI refining was set at 1000, 1500, and 2000, respectively. Seedling pots were prepared using the refined *Miscanthus* pulp as the primary fiber source. To enhance appearance and compressive strength, 10%, 15%, and 20% (*w*/*w*) of *Miscanthus* stalks (particle size of 20–30 mesh) were added to replace the same amount of refined *Miscanthus* pulp accordingly.

The preparation of seedling pots was conducted via the wet foaming method according to the previous report [30] with slight modifications. In detail, a total of 28 g (dry weight) of *Miscanthus* pulp fibers and *Miscanthus* stalks were mixed and dispersed in 1000 mL of deionized water to form a uniform suspension with mechanical stirring. Alkyl polyglycoside non-ionic surfactant was used as a foaming agent. After the foaming agent (the dosage w-as 0.01% based on the total dry weight of *Miscanthus* fibers and stalks) was incorporated, the mixture was continuously stirred until a stable and uniform foam slurry formed. The foamed slurry was then transferred to a bottomless molding die equipped with a 50-mesh wire screen at the bottom. The mold shape was designed according to the size of the seedling pot. After draining the excess filtrate at room temperature for 15 min, the wet foam was pressed to the preset height to get the wet foam pot, which was further oven-dried at 50 °C for 24 h. The pot samples were named as X%-Y-Z% RM, in which X represents the dosage of (NH_4_)_2_SO_3_ in pretreatment, Y is the revolution number of PFI mechanical refining, and Z represents the added amount of *Miscanthus* stalk. For example, pots made from *Miscanthus* pulp fibers pretreated with 15% (NH_4_)_2_SO_3_ and post-refined with revolution numbers of 1000 are denoted as 15%-1000, while the corresponding pots containing 10% *Miscanthus* stalks are labeled as 15%-1000-10% RM. Finally, a waterproof coating was applied by brushing wood wax oil uniformly onto the pot surface, and the coating amount was 0.02 g/cm^2^. After coating, the seedling pots were naturally dried at room temperature.

### 2.4. Characterization

The chemical components of *Miscanthus* before and after pretreatment were analyzed according to the standard National Renewable Energy Laboratory (NREL) procedures (NREL/TP-510-42619 for extractives; NREL/TP-510-42618 for structural carbohydrates and lignin). The pulping degree and water retention value of the refined *Miscanthus* pulp were determined as mentioned in the Supplementary Files. *Miscanthus* samples before and after pretreatment were also characterized by scanning electron microscopy (SEM), X-ray diffraction (XRD), Fourier transform infrared spectroscopy (FTIR), etc., respectively. In addition, the compressive strength, water resistance, biodegradability, and applicability of the *Miscanthus* seedling pot were also tested accordingly. All the characterization details are shown in the Supplementary Documents.

In this work, all the experiments of chemical component analysis of *Miscanthus*, pretreatment, preparation of seedling pots, and the sample characterizations were carried out at least three times, and the average values with standard deviations were reported accordingly.

## 3. Results and Discussion

### 3.1. Optimization of Alkaline Sulfite Pretreatment Conditions

**Figure 2:**
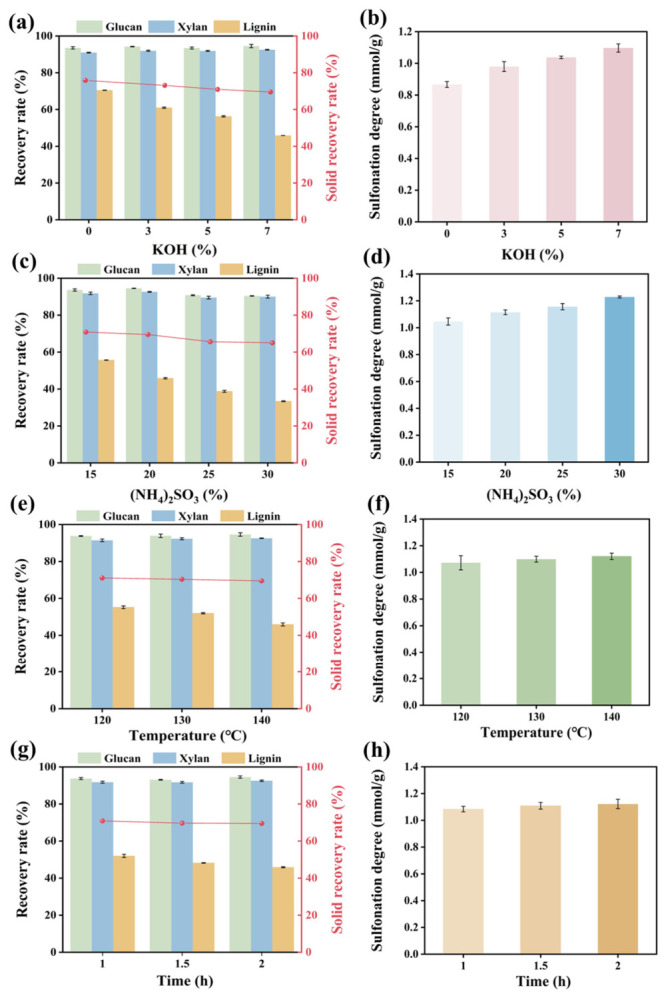
The effects of different pretreatment conditions on the effectiveness of pretreatment in terms of solid recovery rate, the recovery rate of glucan/xylan/lignin, and *DS* of removed lignin. (**a**,**b**) KOH dosage; (**c**,**d**) (NH_4_)_2_SO_3_ dosage; (**e**,**f**) temperature; (**g**,**h**) time.

As shown in Figure 1, alkaline sulfite pretreatment of *Miscanthus* was carried out to separate the cellulosic fibers and lignin. Compared to sulfite pretreatment under neutral conditions, alkali can promote sulfonation of lignin, enhancing lignin removal [31]. Therefore, in this work, a small amount of KOH was added to the sulfite pretreatment. In this case, the spent liquor of pretreatment containing K is also suitable to be used as fertilizer [32]. Herein, the effectiveness of sulfite pretreatment of *Miscanthus* is showcased in Figure 2, based on the single-factor influence experiment.

#### 3.1.1. KOH Dosage

The effects of KOH dosage on the pretreatment effectiveness are presented in Figure 2a,b, and Table S1, and other pretreatment conditions were fixed (20 wt.% (NH_4_)_2_SO_3_, 140 °C for 2 h, solid–liquid ratio of 1:10). As can be seen, when KOH dosage increased, the content of glucan gradually increased from 44.88 ± 0.24% to 49.45 ± 0.36%, while the content of xylan increased from 19.54 ± 0.02% to 21.66 ± 0.01% and the recovery rates of both remained at a relatively high level. Additionally, the increase in KOH dosage led to the gradual dissolution of lignin, and the lignin content decreased from 17.41 ± 0.05% to 12.34 ± 0.04% as the dosage of KOH increased from 0 to 7%. The corresponding rate of lignin removal reached 54.14 ± 0.02% from 29.49 ± 0.09%. Meanwhile, the *DS* of lignin increased from 0.87 ± 0.02 mmol/g to 1.10 ± 0.03 mmol/g (Figure 2b). This phenomenon occurred because KOH broke the ester bonds between lignin and carbohydrates [33], promoted the reaction between (NH_4_)_2_SO_3_ and lignin, introduced more sulfonic groups, and thus enhanced the hydrophilicity and solubility of lignin, thereby facilitating lignin removal. Consequently, a 7 wt.% KOH dosage was selected for the subsequent pretreatment. Further increase in KOH is not needed to avoid severe degradation of carbohydrates and save pretreatment cost.

#### 3.1.2. (NH_4_)_2_SO_3_ Dosage

The effects of (NH_4_)_2_SO_3_ dosage on the pretreatment effectiveness are given in Figure 2c,d, and Table S2 (other factors were fixed as: 7 wt.% KOH, 140 °C for 2 h, solid–liquid ratio of 1:10). As the dosage of (NH_4_)_2_SO_3_ increased from 15 wt.% to 30 wt.%, the lignin content gradually decreased to 9.61 ± 0.15%, achieving a maximum removal rate of 66.59 ± 0.23%. This result was attributed to the fact that the increase in sulfite dosage could lead to more sulfonic groups being introduced into the lignin macromolecules, thus promoting the dissolution of lignin. Correspondingly, the *DS* of lignin in spent liquor increased from 1.05 ± 0.03 mmol/g to 1.23 ± 0.01 mmol/g (Figure 2d). In addition, although the contents of glucan and xylan gradually increased, their recovery rates decreased accordingly. To avoid over-degradation of carbohydrates, the suitable (NH_4_)_2_SO_3_ dosage was selected as 20 wt.%.

#### 3.1.3. Temperature

Temperature influenced the reaction rate and mass transfer efficiency of pretreatment and constituted one of the most critical factors affecting the pretreatment outcome. For instance, an increase in temperature reduced the viscosity of the liquid and caused pore expansion in wood fibers [34], thereby facilitating the penetration of the reaction liquid into the dense microstructure of biomass. The influence of temperature on the pretreatment effectiveness is shown in Figure 2e,f, and Table S3. It can be seen that as the temperature increased from 120 to 140 °C (under other conditions of 7 wt.% KOH, 20 wt.% (NH_4_)_2_SO_3_, 2 h, solid–liquid ratio of 1:10), the lignin content decreased from 14.55 ± 0.16% to 12.37 ± 0.22%, with the rate of lignin removal reaching 54.09 ± 0.85%. Correspondingly, the *DS* of the removed lignin also rose from 1.07 ± 0.05 mmol/g to 1.12 ± 0.02 mmol/g (Figure 2f). Meanwhile, both the contents and recovery rates of glucan and xylan exhibited a gradual increase. The recovery rate of glucan reached 94.59 ± 0.96% from 93.8 ± 0.24%, while the recovery rate of xylan rose from 91.46 ± 0.71% to 92.57 ± 0.15%. Therefore, 140 °C was selected as the suitable pretreatment temperature. Further increase in temperature is not needed to save energy costs and avoid the severe decomposition of (NH_4_)_2_SO_3_.

#### 3.1.4. Time

The pretreatment time is related to whether the pretreatment is sufficient. Along with factors such as reagent dosage and temperature, it jointly affects the effect of pretreatment. As depicted in Figure 2g,h, and Table S4, with the extension of reaction time from 1 to 2 h, the lignin content decreased to 12.35 ± 0.07%, while the lignin removal rate increased from 47.99 ± 0.87% to 54.13 ± 0.30%. Correspondingly, the *DS* of the removed lignin rose from 1.08 ± 0.02 mmol/g to 1.12 ± 0.03 mmol/g (Figure 2h). Also, both the recovery rates of glucan and xylan were basically steady when the pretreatment time increased from 1 to 2 h, indicating no clear degradation of carbohydrates. Consequently, 2 h was chosen as the suitable pretreatment time.

#### 3.1.5. Solid–Liquid Ratio

In the pretreatment process, the solid–liquid ratio directly determined the actual concentration of reagent and mass transfer. The impact of solid–liquid ratio on the pretreatment effectiveness is presented in Figure S1 and Table S5 (other pretreatment conditions were 7 wt.% KOH, 20 wt.% (NH_4_)_2_SO_3_, and 140 °C for 2 h). As the solid–liquid ratio decreased from 1:6 to 1:12, both the content and recovery rate of lignin exhibited a trend of initially decreasing and then increasing. The highest lignin removal (56.45 ± 0.43%) and lowest lignin content (11.86 ± 0.11%) were achieved at the solid–liquid ratio of 1:8, and the corresponding *DS* of removed lignin reached a maximum of 1.13 ± 0.02 mmol/g (Figure S1b). Meanwhile, the corresponding highest recovery rates of glucan and xylan were 94.90 ± 0.41% and 93.89 ± 0.30%, respectively. When the solid–liquid ratio was reduced to 1:12, the contents of glucan and xylan, as well as the lignin removal rate, all decreased, due to the over-dilution of reagents. Consequently, 1:8 was selected as the suitable solid–liquid ratio.

Collectively, according to the single-factor influence experiment of alkaline sulfite pretreatment of *Miscanthus*, the suitable pretreatment conditions were: 7 wt.% KOH, 20 wt.% (NH_4_)_2_SO_3_, 140 °C for 2 h, and a solid–liquid ratio of 1:8.

### 3.2. The Influence of Pretreatment on Miscanthus Structure

**Figure 3:**
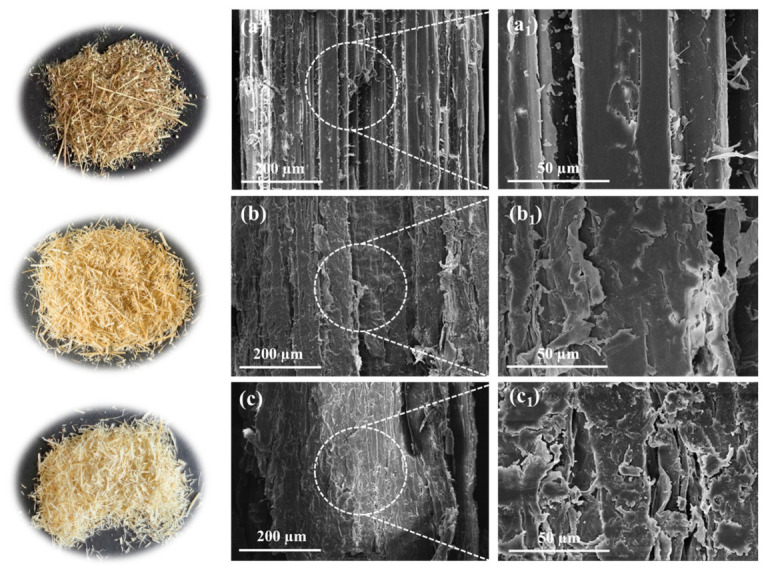
Photos (**left**) and SEM images (**right**) of the *Miscanthus* before and after pretreatment. (**a**) Raw materials; (**b**) 7 wt.% KOH + 20 wt.% (NH_4_)_2_SO_3_; (**c**) 7 wt.% KOH + 30 wt.% (NH_4_)_2_SO_3_. (**a_1_**) enlarged SEM image of the marked part of image (**a**); (**b_1_**) enlarged SEM image of the marked part of image (**b**); (**c_1_**) enlarged SEM image of the marked part of image (**c**).

The morphological changes of *Miscanthus* before and after pretreatment were observed. As shown in Figure 3, the raw material of *Miscanthus* is yellowish-brown. After pretreatment with 7 wt.% KOH and 20 wt.% (NH_4_)_2_SO_3_, the color of *Miscanthus* lightened. This change was due to the removal of lignin. As the dosage of (NH_4_)_2_SO_3_ increased, more lignin was removed and dissolved in the spent liquor of pretreatment, leading to a lighter colour of the pretreated *Miscanthus*. Moreover, the SEM images showed that, prior to pretreatment, the surface of *Miscanthus* appeared relatively smooth (Figure 3a). After alkaline sulfite pretreatment, the surface of *Miscanthus* fibers became rougher and looser because of the partial removal of lignin and extractives. Clearly, the damage and roughness of the surface of *Miscanthus* fibers increased with the increase of (NH_4_)_2_SO_3_ dosage (Figure 3b,c), which was in agreement with the component changes shown in Figure 2c and Table S2.

**Figure 4:**
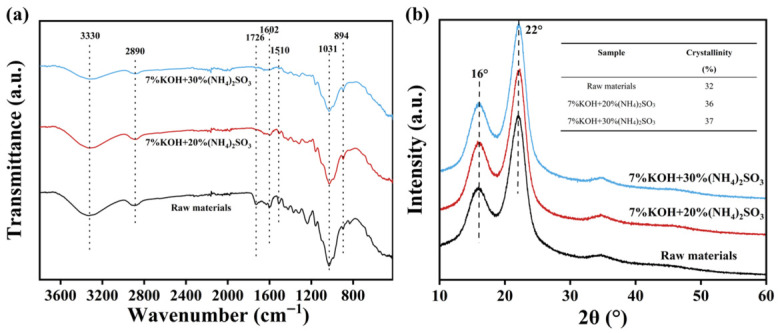
FTIR spectra (**a**) and XRD patterns (**b**) of *Miscanthus* before and after pretreatment.

Figure 4a displays the FTIR spectra of *Miscanthus* before and after pretreatment. The absorption band at 3330 cm^−1^ is attributed to -OH stretching vibrations, while the peak at 2890 cm^−1^ corresponds to the C-H stretching in methylene groups [35]. The intensity of the peak at 1726 cm^−1^, assigned to C=O stretching of acetyl groups in hemicellulose [36], which weakened with the increase of pretreatment degree, indicating a certain degree of hemicellulose degradation. The bands at 1602 cm^−1^ and 1510 cm^−1^, associated with aromatic skeletal vibrations in lignin [37], also diminished, reflecting lignin removal after pretreatment. The peaks at 1031 cm^−1^ are ascribed to C-O-C and C-O stretching vibrations [35], and the signal at 894 cm^−1^ is characteristic of β-(1,4)-glycosidic linkages [38].

Figure 4b shows the changes in the crystal structure and crystallinity of *Miscanthus* before and after pretreatment. The results indicated that *Miscanthus* has two diffraction peaks near 16° and 22°, suggesting that the cellulose in *Miscanthus* has a type Iβ crystal structure [39]. Compared with the raw materials, the intensification of the two diffraction peaks increased after pretreatment with 7 wt.% KOH and 20 wt.% (NH_4_)_2_SO_3_. This phenomenon was due to the partial removal of amorphous components such as lignin, hemicellulose, and extractives. Furthermore, this pretreatment may also exert a small destructive effect on the amorphous region of cellulose. Consequently, the relative proportion of the crystalline cellulose increased. Therefore, the crystallinity of *Miscanthus* increased after the alkaline sulfite pretreatment, as shown in Figure 4b.

### 3.3. Impact of Post-Mechanical Refining on the Properties of Miscanthus Pulp and Seedling Pots

**Figure 5:**
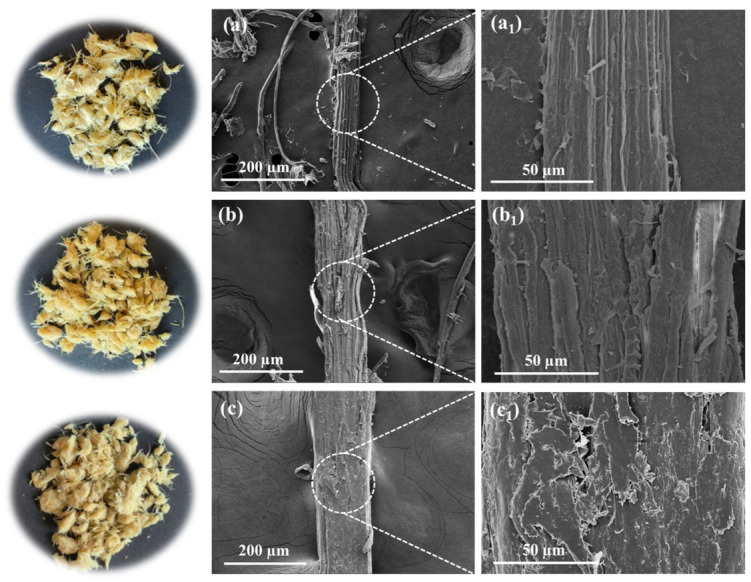
Appearance (**left**) and SEM (**right**) images of *Miscanthus* fibers after pretreatment with 20 wt.% (NH_4_)_2_SO_3_ and PFI refining with different revolution numbers: (**a**) PFI revolution number of 1000; (**b**) PFI revolution number of 1500; (**c**) PFI revolution number of 2000. (**a_1_**) the enlarged SEM image of the marked part of image (**a**); (**b_1_**) the enlarged SEM image of the marked part of image (**b**); (**c_1_**) the enlarged SEM image of the marked part of image (**c**).

As known [40,41], post mechanical refining (after chemical pretreatment) could further modify the properties of cellulosic fibers for improving fiber bonding potential and strength of paper products, by increasing internal fibrillation/cell wall delamination, softening fibers, generating fines, and increasing specific surface area of fibers and fines. Hence, in this work, before preparing seedling pots, the fractionated cellulosic pulp from sulfite pretreatment of *Miscanthus* was subjected to post PFI refining, and the impact of PFI refining on the properties of *Miscanthus* pulp fibers was evaluated. The appearance and SEM images of *Miscanthus* pulp under varying pretreatment conditions and different revolution numbers of PFI refining are presented in Figure 5 and Figure S2. After sulfite pretreatment without post PFI refining, *Miscanthus* fibers still looked relatively rigid (Figure 3), while the pretreated *Miscanthus* fibers became clearly softened after post PFI refining (Figure 5). SEM images (Figure 5 and Figure S2) show that, at a lower revolution number (1000), the surfaces of the fibers appeared relatively smooth, exhibiting only a few grooves and minor mechanical damage. As the revolution number increased, the surfaces of the fibers began to experience wear, and internal fibrillation/fiber delamination occurred, resulting in a rougher texture. When the revolution number was 2000, the surface wear of the fibers was most pronounced, displaying a highly rough morphological characteristic for both the samples with 15 wt.% and 20 wt.% (NH_4_)_2_SO_3_.

**Figure 6:**
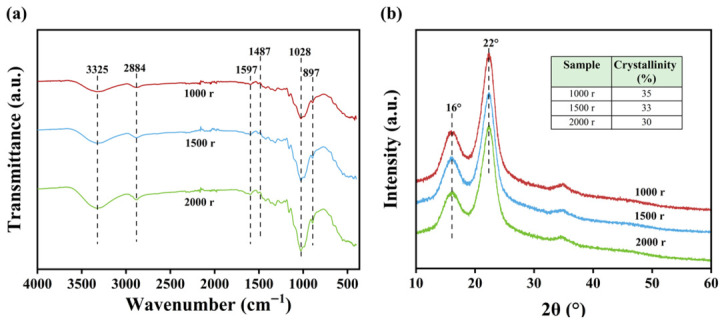
FTIR spectra (**a**) and XRD patterns (**b**) of *Miscanthus* fibers after pretreatment with 20 wt.% (NH_4_)_2_SO_3_ and PFI refining with different revolution numbers.

Figure 6a and Figure S3a present the FTIR spectra of *Miscanthus* pulp. The absorption peak at 3325 cm^−1^ corresponded to the stretching vibration of the -OH group [35]. As the revolution number of PFI refining increased, the intensity of this absorption peak gradually strengthened, which was attributed to the increased exposure of hydroxyl groups on the fiber surface due to the fibrillation/fiber delamination. The absorption peak at 1028 cm^−1^ corresponds to the stretching vibrations of the C-O-C and C-O bonds in alcohols [35]. Notably, the intensity of this absorption peak exhibited a clear change with the increase in the revolution number of PFI refining. This phenomenon may stem from the change in the surface structure of the fibers during the refining process: fine fibers increased the specific surface area of the fibers, leading to an enhanced infrared absorption of these functional groups.

Figure 6b and Figure S3b present the XRD patterns and crystallinity index of *Miscanthus* pulp after post-PFI refining. The diffraction peaks near 14°, 16°, and 22° correspond to the cellulose Iβ crystalline structure [42], confirming that the refining process did not alter the cellulose crystal form. Under identical sulfite pretreatment conditions, the peak intensities and crystallinity at these two positions decreased markedly as the degree of PFI refining increased. This phenomenon was due to the partial damage of the crystalline region of cellulose during mechanical refining action [43], which was in agreement with the gradual decrease of crystalline index (Figure 6b and Figure S3b).

**Figure 7:**
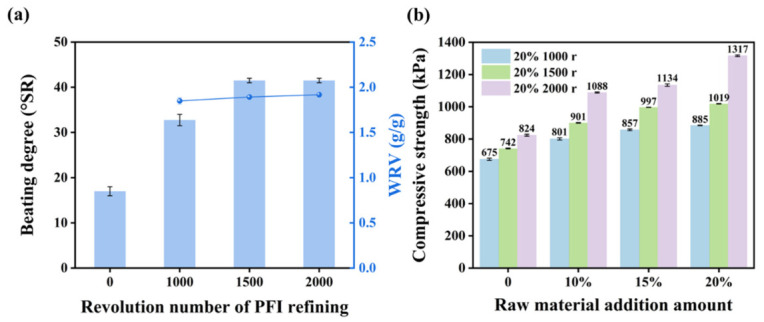
The beating degree and water retention value of *Miscanthus* pulp under different revolution numbers of PFI refining (**a**) and compressive strength of *Miscanthus* seedling pots (**b**) with the compressive strain of 80%. (The used pulp was the pretreated *Miscanthus* with 20 wt.% (NH_4_)_2_SO_3_).

The beating degree reflects the water retention value (WRV) of the pulp, indicating fiber porosity and delamination after refining. Higher beating degree values correspond to greater WRV, increased fiber porosity, and enhanced stratification [40]. As shown in Figure S4a, for the one after sulfite pretreatment with 15 wt.% (NH_4_)_2_SO_3_, both beating degree and WRV increased with refining intensity: when the PFI refining revolution number was 0, the beating degree of *Miscanthus* was 14 ± 1 °SR; as the revolution number of PFl refining increased from 1000 to 2000, the beating degree rose from 21 ± 1 °SR to 29 ± 1 °SR, and the corresponding WRV increased from 1.53 ± 0.01 g/g to 1.81 ± 0.03 g/g. This result was because the mechanical action of PFI refining refined the fibers, increased the specific surface area of the fibers, and formed a dense fiber network [44], and thus enhanced the beating degree and water retention value. However, the presence of residual lignin with a relatively high content limited the fibrillation of fibers, resulting in a relatively low overall beating degree. For the one after sulfite pretreatment with 20 wt.% (NH_4_)_2_SO_3_, the beating degree and WRV of the pulp increased with the increase of revolution number from 1000 to 1500, and tended to be stable as the revolution number further increased to 2000. Because of the relatively low lignin content in the pulp with 20 wt.% (NH_4_)_2_SO_3_, the fibers were susceptible to fibrillation, leading to a high pulping degree and WRV. When the PFI refining revolution number was 0, the corresponding beating degree of *Miscanthus* was 17 ± 1 °SR; when the revolution number of PFI refining increased from 1000 to 2000, the corresponding beating degree increased from 33 ± 1 °SR to 42 ± 1 °SR, and the WRV rose from 1.85 ± 0.04 g/g to 1.92 ± 0.01 g/g accordingly (Figure 7a).

After post-PFI refining, the refined fibers were used to prepare seedling pots via the wet foaming technique [28]. The compressive strength of *Miscanthus* seedling pots is presented in Figure 7b and Figure S4b. Under identical pretreatment conditions, the compressive strength of pot samples increased with the increase of PFI revolution numbers. This result was attributed to the enlarged specific surface area of the refined fibers and greater exposure of hydroxyl groups after intensive refining, which promoted the formation of more hydrogen bonds and enhanced inter-fiber bonding during drying and shaping [45]. It is noteworthy that, for the ones with 20 wt.% (NH_4_)_2_SO_3_, although the beating degrees of *Miscanthus* pulp were similar when the revolution number of PFl refining was 1500 and 2000, respectively, there was an obvious disparity in the compressive strength of the corresponding seedling pots. The compressive strength of the 20%-2000 sample was 823.85 ± 5.99 kPa, whereas the compressive strength of the 20%–1500 sample was 741.62 ± 2.29 kPa. This result was because the pulp at 2000 r experienced more fibrillation of fibers, leading to a larger specific surface area for the fibers and thus enhancing the bonding strength between more refined fibers. Consequently, the compressive strength of the 20%-2000 sample was relatively higher.

In addition, to enhance appearance and compressive strength, 10%, 15%, and 20% (*w*/*w*) of raw *Miscanthus* stalks (particle size of 20–30 mesh) without pretreatment were directly added to replace the same amount of refined *Miscanthus* pulp accordingly. Compared with the samples without the addition of raw *Miscanthus* stalks, the compressive strength of the samples was clearly enhanced after the addition of raw *Miscanthus* stalks, and the strength increased with the increase in the amount of *Miscanthus* stalks added. This result was due to the addition of *Miscanthus* stalks, which introduced harder fibers, establishing a supportive framework within the pulp. Simultaneously, the highly refined pulp exhibited enhanced inter-fiber binding, enabling complete encasement of the *Miscanthus* stalks and reinforcing the framework. The combined action of these two factors (i.e., post-PFI refining and the addition of raw *Miscanthus* stalks) led to a rise in the compressive strength of the seedling pots. The compressive strength of the 20%-2000-20%RM sample was the highest, reaching 1316.62 ± 4.89 kPa, thereby confirming this inference.

### 3.4. Water-Resistant Properties of the Seedling Pot

**Figure 8:**
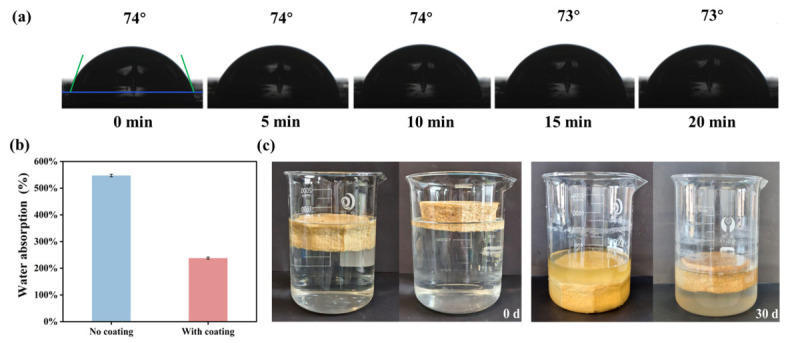
Water resistance of the prepared seedling pots before and after surface coating with a coating dose of 0.02 g/cm^2^. (The photos of the water angle changes on the sample surface over time (**a**); the water absorption of the 20–2000-20%RM sample after 30 min (**b**); photos of the *Miscanthus* seedling pot after being soaked in water for 30 days ((**c**), left: uncoated; right: coated).

Wood wax oil, as a natural coating composed primarily of linseed oil, sunflower oil, beeswax, and natural resins, exhibits film-forming ability and can penetrate effectively into wooden substrates. It is environmentally benign throughout its lifecycle, as it is free from pungent odors, solvents (e.g., formaldehyde, benzene, toluene), and heavy metals (such as lead or manganese) [46]. To improve the water resistance of the prepared seedling pots, surface coating of wood wax oil was carried out with a coating dose of 0.02 g/cm^2^. As shown in Figure 8a, the water contact angle of the coated seedling pot surface was 74°. Also, during a continuous observation period of up to 30 min, the water contact angle was very stable with only a slight decrease of 1°. However, for the one without a surface coating, the water droplets were immediately absorbed due to the hydrophilic and porous surface of the pot samples. Correspondingly, the water absorption of the uncoated pot samples was as high as 547.38 ± 4.82%, which was about 130% higher compared to the coated sample (Figure 8b). This reduction was attributed to the relatively hydrophobic surface formed by the coating, which partially sealed the surface pores and restricted the penetration of water. However, even after the coating treatment, the water absorption of the sample remained relatively high, indicating that the waterproof mechanism of this coating might have involved the construction of a hydrophobic waxy layer on the sample surface rather than a completely impermeable sealing layer. Furthermore, the sample itself exhibited a relatively high hydrophilicity, which, to some extent, contributed to the elevated water absorption, even after being coated with wood wax oil.

The water resistance of the samples was further assessed through a 30-day immersion test (Figure 8c). At 0 d, the uncoated samples sank below the water surface due to their porous and hydrophilic nature. However, the coated sample can float in water with most of the sample above the water surface, and even keeps floating after 30 days. This phenomenon was because the wood wax oil coating not only served a certain waterproof function but also slowed down the penetration rate of water. This relatively good water-resistant surface of the pots is suitable for their use as seedling pots.

### 3.5. Planting Experiment

**Figure 9:**
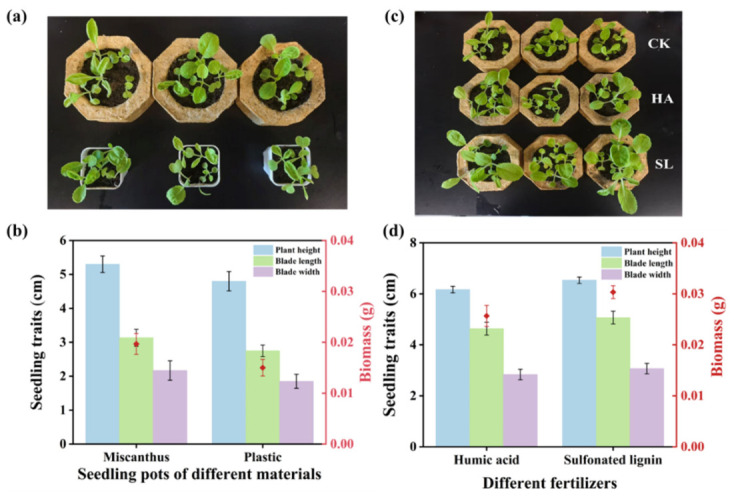
Comparison of planting experiment of Chinese cabbage seedlings in different seedling pots ((**a**): growth photos; (**b**): plant sizes) and under different fertilization conditions ((**c**): growth photos; (**d**): plant sizes) with 10-day growth.

Compared with plastic seedling pots, the Chinese cabbage seedlings cultivated in *Miscanthus* seedling pots demonstrated certain advantages in multiple growth indicators (Figure 9a,b). Under the same cultivation management conditions, the leaf morphology development of Chinese cabbage seedlings in the *Miscanthus* seedling pots was more vigorous. The leaf length and width measured as 3.13 ± 0.25 cm and 2.17 ± 0.29 cm, respectively, both surpassing those for the plastic seedling pots group. Meanwhile, the seedling height in the *Miscanthus* seedling pots reached 5.30 ± 0.24 cm, which represented a 10% increase over the latter, thereby demonstrating more vigorous elongation growth of the above-ground vegetative body. Ultimately, these morphological advantages led to a substantial accumulation of biomass (dry weight), with the biomass of the *Miscanthus* seedling pots group representing a 33% increase compared with the plastic seedling pots group. These results indicated that the seedling pots developed from *Miscanthus* fulfilled the functional requirements for seedling cultivation, thereby demonstrating their viability as a bio-based alternative to conventional plastic seedling pots.

This experiment also evaluated the effect of SL as a fertilizer on the growth of cabbage seedlings (Figure 9c,d). Compared to the equivalent amount of HA, the seedling height in the SL treatment group reached 6.53 ± 0.12 cm, representing a 6% increase over the HA treatment group. The lengths and widths of the leaves measured as 5.07 ± 0.25 cm and 3.07 ± 0.21 cm, respectively, reflecting increases of approximately 10% and 8% compared to the HA treatment group. These results indicated that the photosynthetic area was expanded more fully, thereby establishing a solid foundation for subsequent material accumulation. Consequently, the biomass (dry weight) of the SL treatment group also increased by 15% relative to that of the HA treatment group. This result can be attributed to the fact that the molecular structure of SL is rich in phenolic hydroxyl groups, carboxyl groups, and sulfonic groups, which gives it strong cationic chelating ability, which can effectively fix ammonium nitrogen, potassium ions, and trace elements in soil. In addition, the good water solubility and surface activity of SL can also improve the soil environment and promote root development and nutrient uptake. Therefore, SL derived from sulfite pretreatment of *Miscanthus* can be potentially used as a new type of functional fertilizer in promoting the growth of vegetable seedlings, although more evaluation (e.g., field experiment) is needed.

### 3.6. Biodegradability

**Figure 10:**
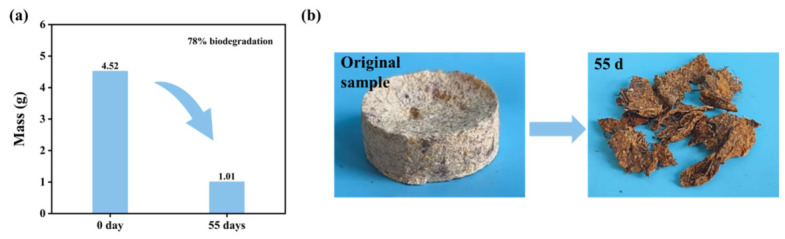
Biodegradability of *Miscanthus* seedling pots without coating (the weight changes of the sample under composting degradation environment (**a**); the appearance of the foam sample before and after degradation (**b**)).

**Figure 11:**
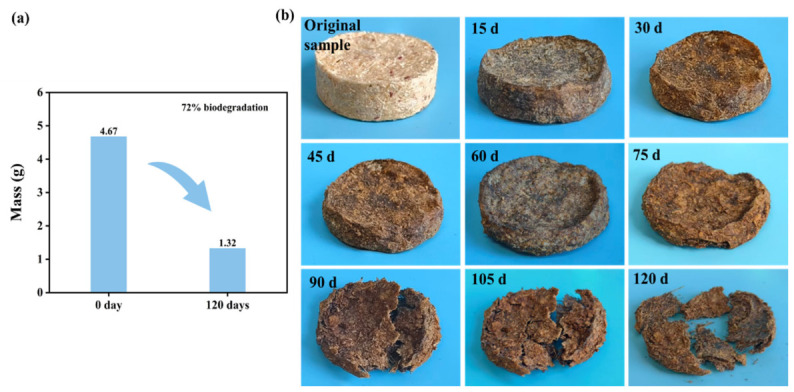
Biodegradability of *Miscanthus* seedling pots with coating (the weight changes of the sample under composting degradation environment (**a**); the appearance of the foam sample before and after degradation (**b**)).

To evaluate the biodegradability of the *Miscanthus* seedling pots, composting degradation experiments were carried out using miniaturized samples with the same formula as the seedling pots. The uncoated sample was degraded into fragments on the 55th day (Figure 10b), exhibiting a degradation rate of 78% (Figure 10a). The coated sample was not degraded into fragments until day 120 of the composting experiment (Figure 11b), suggesting that the application of wood wax oil postponed the degradation process. As indicated in Figure 11a, after 120 days of the composting experiment, the degradation rate of the coated sample reached 72%. Concurrently, the sample’s shape diminished, its color darkened, and mold spots appeared on the surface (Figure 11b). This phenomenon occurred due to the compost soil’s richness in bacteria and fungi, which facilitated the degradation of the samples. The above results demonstrated that the seedling pots exhibited a certain degree of biodegradability.

## 4. Conclusions

This study established a new approach for the better use of *Miscanthus* based on alkaline sulfite pretreatment for preparing biodegradable seedling pots and lignin-based fertilizer. According to the single-factor influence experiment, the suitable conditions of alkaline sulfite pretreatment were 7 wt.% KOH, 20 wt.% (NH_4_)_2_SO_3_, solid–liquid ratio of 1:8, and 140 °C for 2 h. Under such conditions, the recovery rate of cellulosic solid was 68.7% without severe degradation of carbohydrates, the rate of delignification was 56%, and the *DS* of removed lignin reached 1.13 mmol/g. After pretreatment, the fractionated cellulosic fibers were further mechanically refined to increase fibrillation/fiber delamination and the specific surface area of fibers. The refined fibers were subjected to the preparation of seedling pots via the wet foaming technique. It was found that suitable post-mechanical refining, lignin removal, and partial addition of raw *Miscanthus* stalks were beneficial to strengthen the resultant seedling pots, and the maximum compressive strength of the prepared seedling pots was 1317 kPa. Also, the surface coating with wood wax imparted certain waterproof characteristics to the *Miscanthus* seedling pots, which were suitable for practical use for seedling cultivation. Additionally, the coated *Miscanthus* seedling pots were biodegradable, which is good for reducing plastic pollution. In the planting experiment, cabbage seedlings cultivated in *Miscanthus* seedling pots exhibited superior performance in key metrics such as leaf length and width, root height, and total biomass, compared to the fossil-based pots. Furthermore, the separated SL can be used as fertilizer for cabbage cultivation, and its efficacy surpassed commercial HA under the same cultivation conditions. Therefore, the established whole process for the full utilization of *Miscanthus* is green and sustainable.

## Data Availability

The original contributions presented in this study are included in the article/Supplementary Materials. Further inquiries can be directed to the corresponding author.

## Supplementary Materials

The following supporting information can be downloaded at: https://www.mdpi.com/article/10.3390/polym18101181/s1. Figure S1: The influence of solid-liquid ratios on the pretreatment effectiveness of *Miscanthus*; Figure S2: Appearance (left) and SEM images (right) of *Miscanthus* fibers after pretreatment with 15 wt.% (NH_4_)_2_SO_3_ pretreatment and PFI refining with different revolution numbers; Figure S3: FTIR spectra (a) and XRD patterns (b) of *Miscanthus* fibers after pretreatment with 15 wt.% (NH_4_)_2_SO_3_ and PFI refining with different revolution numbers; Figure S4: Beating degree and water retention value of *Miscanthus* fibers under different revolution numbers of PFI refining (a) and the compressive strength of *Miscanthus* seedling pots with the compressive strain of 80% (b); Figure S5: Stress-strain curves of *Miscanthus* seedling pots prepared using the pulp with 15 wt.% (NH_4_)_2_SO_3_ and different PFI revolution numbers; Figure S6: Stress-strain curves of *Miscanthus* seedling pots prepared using the pulp with 20 wt.% (NH_4_)_2_SO_3_ and different PFI revolution numbers; Table S1: The influence of KOH dosages on the component changes of *Miscanthus* after pretreatment; Table S2: The influence of (NH_4_)_2_SO_3_ dosage on the components changes of *Miscanthus* after pretreatment; Table S3: The influence of temperature on the components changes of *Miscanthus* after pretreatment; Table S4: The influence of time on the components changes of *Miscanthus* after pretreatment; Table S5: The influence of solid-liquid ratio on the components changes of *Miscanthus* after pretreatment.

## Abbreviations

The following abbreviations are used in this manuscript:

SL	sulfonated lignin
HA	humic acid
SEM	scanning electron microscopy
XRD	X-ray diffraction
FTIR	Fourier transform infrared spectroscopy
WRV	water retention value
*DS*	degree of sulfonation
