# Supplementary material for: Sustainable Strategies for Full Use of Miscanthus: Biodegradable Seedling Pots and Lignin-Based Fertilizers
Source: Polymers (Basel). 2026 May 12;18(10):1181. doi: 10.3390/polym18101181 (PMC13211002; doi:10.3390/polym18101181)
Supplement: Supplementary file 1 [file polymers-18-01181-s001.zip › polymers-4282769-supplementary.pdf]

Supplementary Material

Sustainable Strategies for Full Use of *Miscanthus*: Biodegradable Seedling Pots and Lignin-Based Fertilizers

Jiyu Guan [1, 2, #], Chao Liu [2, #], Guang Yu [2], Mohammad Ali Asadollahi [3], Chunxiang Fu [2], Wangda Qu [1, *], Bin Li [2, *]

[1] College of Life Sciences, Qingdao Agricultural University, Qingdao 266109, China.

[2] Qingdao New Energy Shandong Laboratory, System Integration Engineering Center, Qingdao Institute of Bioenergy and Bioprocess Technology, Chinese Academy of Sciences, Qingdao, Shandong 266101, China

[3] Department of Biotechnology, Faculty of Biological Science and Technology, University of Isfahan, Isfahan 81746-73441, Iran

* Correspondence: wqu@qau.edu.cn (W. Q.); e-mail: libin@qibebt.ac.cn (B. L.)

[#] These authors contributed equally to this work.

Analysis and Characterization

Component analysis of *Miscanthus* before and after pretreatment was determined according to the NREL procedure (NREL/TP-510-42619 for extractives; NREL/TP-510-42618 for structural carbohydrates and lignin). The high-performance liquid chromatography (HPLC) system (Model 1200, Agilent, USA) used in this work was equipped with a Berid Aminex HPX-87H column and a refractive index detector. The temperature of the chromatographic column was 55 °C, the mobile phase was 0.005 M H_2SO_4, and the flow rate was 0.6 mL/min. The recovery rate of cellulose, hemicellulose, lignin, rate of lignin removal, and recovery rate of solid were evaluated by the following equations:

R_{solid} (%)= [Mass of pretreated dry *Miscanthus* (g)/ Mass of initial dry *Miscanthus* (g)] × 100 (S-1)

R_{glucan} (%) = (R_{solid} × Glucan content in pretreated *Miscanthus* / Glucan content in raw *Miscanthus*) × 100 (S-2)

R_{xylan} (%) = (R_{solid} × Xylan content in pretreated *Miscanthus* / Xylan content in raw *Miscanthus*) × 100 (S-3)

R_{lignin} (%) = （R_{solid} × Lignin content in pretreated *Miscanthus* / Lignin content in raw *Miscanthus*） × 100 (S-4)

$R_{delignification}$ (%) = 1 − R_{lignin} (S-5)

Where, R_{solid} is the recovery rate of solid, R_{glucan} is the recovery rate of glucan, R_{xylan} is the recovery rate of xylan, R_{lignin} is the recovery rate of lignin, and $R_{delignification}$ is the rate of delignification.

The degree of sulfonation (*DS*) of lignin was measured according to the previously reported procedure[1]. In detail, lignin solution was sequentially passed through anion-exchange (717) and cation-exchange (732) resins, which were pretreated with NaOH (1-3 mol/L) and HCl (1-3 mol/L), respectively, and then rinsed to neutrality. The resulting solution was titrated with 0.02 mol/L NaOH to monitor conductivity. The SD of sulfonated lignin was calculated as:

$$DS = \frac{N_{\mathrm{NaOH}} \times V_{\mathrm{NaOH}}}{W_L} \quad \text{(S-6)}$$

Where, *DS* represents the degree of sulfonation of sulfonated lignin (mmol/g),

N_{NaOH} represents the concentration of the NaOH standard solution (mol/L), V_{NaOH} is the consumed volume (mL) of the NaOH standard solution, and W_L is the weight of lignin in the solution.

The micro-morphology of the *Miscanthus* samples was examined using a cold-field emission scanning electron microscope (SEM, S-4800, Hitachi, Japan). Before imaging, the freeze-dried samples were mounted on an aluminum sample stage using conductive tape and sputter-coated with a gold layer for 120 s to enhance conductivity. Attenuated total reflection - Fourier transform infrared spectroscopy (ATR-FTIR, Nicolet 6700 FT-IR spectrometer, Thermo Fisher, USA) was used to characterize the chemical structures of the *Miscanthus* samples, with spectra collected over a wavenumber range from 4000 to 350 cm^{-1}. The crystal structure of the *Miscanthus* samples was analyzed by an X-ray diffractometer (XRD, D8 Advance, Bruker, Germany). The scattering Angle (2θ) was ranged from 10 to 60°, and the scanning speed was 0.5°/min. The crystallinity index (*CrI*) of *Miscanthus* samples was analyzed using the following equation (Segal method):

$$CrI = \frac{I_{200} - I_{am}}{I_{200}} \times 100\% \tag{S-7}$$

Where, I_{200} is the maximum diffraction intensity of the 200 plane, and I_{am} is the minimum diffraction intensity between peaks 101 and 200.

The beating degree (°SR) of the *Miscanthus* pulp fibers was measured using a beating degree tester (DF-8871, AISRY, China) following the international standard (ISO 5267-1:1999). Briefly, 1000 ± 5 mL of the slurry suspension was poured into a graduated cylinder, stirred thoroughly, and then transferred into the chamber of the tester. The reading was taken after the drainage reached equilibrium. The water retention value (WRV) was determined in accordance with the international standard (ISO 23714:2014).

The compressive strength of the *Miscanthus* seedling pots was determined using an electronic universal testing machine (CMT 6503, MTS, China) in accordance with the national standard GB 8168-2008. A 5 kN load cell was used, with a crosshead speed of 100 mm/min and a compression strain of 80%. Prior to testing, all samples were

conditioned at 23 °C and 50% relative humidity for 24 h.

The water contact angle of the *Miscanthus* seedling pot samples was measured using a water contact angle measuring instrument (CSSDIC-200S, SINDIN, China) by depositing a 20 μL deionized water droplet on the sample surface. The water resistance of the seedling pot samples was tested by a soaking experiment. The dried sample was placed in a beaker containing 1500 mL of distilled water and left until it reached equilibrium. The samples were immersed for 30 days, during which the morphological changes of the samples were observed. The water absorption (W, %) of the samples was determined after immersing the sample in deionized water for 30 min, calculated as:

$$W = \frac{m_2 - m_1}{m_1} \times 100\% \quad \text{(S-8)}$$

Where, m_1 and m_2 are the weights of the sample before and after immersion, respectively.

A comparative planting experiment was conducted under natural conditions using the prepared seedling pots alongside conventional plastic pots as the control. Each pot was sown with 5-6 Chinese cabbage seeds. An initial watering of 50-100 mL was applied on the first day, followed by supplemental irrigation as needed based on soil moisture levels. During the seedling growth period, natural light exposure was initially maintained at 4-5 h per day (days 0-2) and then gradually increased in accordance with seedling development. The ambient temperature throughout the experiment averaged approximately 25 °C. 0.15 g of the sulfonated lignin (SL) obtained after pretreatment was directly used as fertilizer, which was compared with an equal amount of humic acid (HA) to evaluate the effect of SL fertilizer on the growth of seedlings. After the experiment, 4 representative seedlings from each treatment were randomly selected and washed with deionized water. The samples were blanched in a 105 °C oven for 30 min, then dried at 75 °C until constant weight was achieved, and finally cooled in a desiccator before measuring the dry weight of the samples (which represents the biomass). Additionally, the height of the roots and stems, the length and width of the leaves were measured to evaluate the growth conditions of cabbage seedlings under different conditions. The average value for each test was reported accordingly.

Biodegradability of seedling pot samples was assessed in accordance with the international standard of composting degradation experiment (ISO 20200:2023). Briefly, the waste matrix was obtained by mixing 40% wood chip, 30% rabbit feed, 10% ripe compost, 10% corn starch, 5% saccharose, 4% corn oil, and 1% urea, and then deionized water (DI) was added with a matrix/water ratio of 45/55. Subsequently, the miniaturized *Miscanthus* seedling pot samples with a diameter of 5 cm and a height of 1.5 cm were buried in the soil to a depth of 6 cm. The biodegradation environment was maintained at 58 ± 2 °C, and DI water was supplemented regularly to maintain the moisture content of the compost. Prior to weighing the sample, the waste matrix was meticulously removed from the surface.

The morphological changes of the seedling pot samples were photographically recorded throughout the test. The degradation rate (D, %) was calculated using the following equation:

$$D = \frac{m_1 - m_2}{m_1} \times 100\% \quad \text{(S-9)}$$

Where, m_1 is the initial dry weight of sample (g), and m_2 is the dry weight (g) of the residual sample after degradation experiment.

Table S1 The influence of KOH dosages on the component changes of *Miscanthus* after pretreatment.

**KOH dosage (%, w/w)**	**Raw materials**	**0**	**3**	**5**	**7**
Content of extractives (%)	21.75 ± 0.37	11.02 ± 0.49	10.80 ± 0.10	11.09 ± 0.43	10.87 ± 0.84
Content of glucan (%)	36.37 ± 0.41	44.88 ± 0.24	46.87 ± 0.02	47.91 ± 0.08	49.45 ± 0.36
Content of xylan (%)	16.28 ± 0.16	19.54 ± 0.02	20.47 ± 0.05	21.08 ± 0.10	21.66 ± 0.01
Content of arabinose (%)	0.19 ± 0.03	0.87 ± 0.01	1.02 ± 0.01	1.11 ± 0.02	1.15 ± 0.02
Content of lignin (%)	18.72 ± 0.15	17.41 ± 0.05	15.61 ± 0.07	14.86 ± 0.02	12.34 ± 0.04
Solid recovery rate (%)	100	75.81 ± 0.12	73.13 ± 0.10	70.95 ± 0.54	69.54 ± 0.19
Recovery rate of glucan (%)	\	93.55 ± 0.65	94.25 ± 0.16	93.46 ± 0.56	94.55 ± 0.93
Recovery rate of xylan (%)	\	90.99 ± 0.22	91.95 ± 0.36	91.88 ± 0.27	92.53 ± 0.21
Recovery rate of lignin (%)	\	70.51 ± 0.09	61.00 ± 0.34	56.31 ± 0.37	45.86 ± 0.02
Lignin removal rate (%)	\	29.49 ± 0.09	39.00 ± 0.34	43.69 ± 0.37	54.14 ± 0.02
Lignin sulfonation degree(mmol/g)	\	0.87 ± 0.02	0.98 ± 0.03	1.04 ± 0.01	1.10 ± 0.03

Other pretreatment conditions are 20 wt.% $(NH_4)_2SO_3$, 140 °C, 2 h, solid-liquid ratio was 1:10.

Table S2 The influence of $(NH_4)_2SO_3$ dosage on the components changes of *Miscanthus* after pretreatment.

**$(NH_4)_2SO_3$ dosage (%, w/w)**	**15**	**20**	**25**	**30**
Content of extractives (%)	11.67 ± 0.30	10.86 ± 0.71	9.23 ± 0.48	10.63 ± 0.93
Content of glucan (%)	48.05 ± 0.11	49.47 ± 0.01	50.33 ± 0.22	50.56 ± 0.51
Content of xylan (%)	21.10 ± 0.06	21.70 ± 0.03	22.20 ± 0.02	22.53 ± 0.01
Content of arabinose (%)	1.49 ± 0.03	1.04 ± 0.01	1.02 ± 0.31	1.27 ± 0.05
Content of lignin (%)	14.71 ± 0.09	12.35 ± 0.09	11.06 ± 0.07	9.61 ± 0.15
Solid recovery rate (%)	70.85 ± 0.31	69.51 ± 0.08	65.62 ± 0.48	65.05 ± 0.55
Recovery rate of glucan (%)	93.61 ± 0.62	94.55 ± 0.09	90.80 ± 0.27	90.42 ± 0.15
Recovery rate of xylan (%)	91.82 ± 0.69	92.64 ± 0.24	89.50 ± 0.73	90.04 ± 0.78
Recovery rate of lignin (%)	55.68 ± 0.11	45.85 ± 0.40	38.77 ± 0.53	33.41 ± 0.23
Lignin removal rate (%)	44.32 ± 0.11	54.15 ± 0.40	61.23 ± 0.53	66.59 ± 0.23
Lignin sulfonation degree (mmol/g)	1.05 ± 0.03	1.11 ± 0.02	1.16 ± 0.02	1.23 ± 0.01

Other pretreatment conditions are 7 wt.% KOH, 140 °C, 2 h, solid-liquid ratio was 1:10.

Table S3 The influence of temperature on the components changes of *Miscanthus* after pretreatment.

**Temperature (°C)**	**120**	**130**	**140**
Content of extractives (%)	7.31 ± 0.35	9.45 ± 0.35	9.70 ± 0.51
Content of glucan (%)	48.03 ± 0.01	48.54 ± 0.33	49.50 ± 0.45
Content of xylan (%)	20.96 ± 0.11	21.36 ± 0.06	21.69 ± 0.06
Content of arabinose (%)	1.08 ± 0.20	1.11 ± 0.17	1.19 ± 0.05
Content of lignin (%)	14.55 ± 0.16	13.83 ± 0.14	12.37 ± 0.22
Solid recovery rate (%)	71.03 ± 0.17	70.34 ± 0.22	69.50 ± 0.07
Recovery rate of glucan (%)	93.80 ± 0.24	93.88 ± 0.94	94.59 ± 0.96
Recovery rate of xylan (%)	91.46 ± 0.71	92.31 ± 0.55	92.57 ± 0.15
Recovery rate of lignin (%)	55.19 ± 0.73	51.95 ± 0.35	45.91 ± 0.85
Lignin removal rate (%)	44.81 ± 0.73	48.05 ± 0.35	54.09 ± 0.85
Lignin sulfonation degree (mmol/g)	1.07 ± 0.05	1.10 ± 0.02	1.12 ± 0.02

Other pretreatment conditions are 7 wt.% KOH, 20 wt.% $(NH_4)_2SO_3$, 2 h, solid-liquid ratio was 1:10.

Table S4 The influence of time on the components changes of *Miscanthus* after pretreatment.

Time (h)	1	1.5	2
Content of extractives (%)	9.86 ± 0.02	10.78 ± 0.64	9.71 ± 0.60
Content of glucan (%)	48.11 ± 0.01	48.58 ± 0.50	49.47 ± 0.38
Content of xylan (%)	21.08 ± 0.01	21.42 ± 0.06	21.67 ± 0.07
Content of arabinose (%)	1.16 ± 0.31	1.27 ± 0.08	1.39 ± 0.24
Content of lignin (%)	13.74 ± 0.31	12.95 ± 0.06	12.35 ± 0.07
Solid recovery rate (%)	70.86 ± 0.40	69.69 ± 0.56	69.52 ± 0.08
Recovery rate of glucan (%)	93.73 ± 0.56	93.08 ± 0.21	94.55 ± 0.61
Recovery rate of xylan (%)	91.76 ± 0.54	91.71 ± 0.47	92.55 ± 0.39
Recovery rate of lignin (%)	52.01 ± 0.87	48.23 ± 0.16	45.87 ± 0.30
Lignin removal rate (%)	47.99 ± 0.87	51.77 ± 0.16	54.13 ± 0.30
Lignin sulfonation degree (mmol/g)	1.08 ± 0.02	1.11 ± 0.03	1.12 ± 0.03

Other pretreatment conditions are 7 wt.% KOH, 20 wt.% $(NH_4)_2SO_3$, 140°C, solid-liquid ratio was 1:10.

Table S5 The influence of solid-liquid ratio on the components changes of *Miscanthus* after pretreatment.

Solid-liquid ratio	1：6	1：8	1：10	1：12
Content of extractives (%)	11.55 ± 0.71	11.03 ± 0.55	11.29 ± 0.30	11.38 ± 0.82
Content of glucan (%)	49.36 ± 0.03	50.21 ± 0.18	49.51 ± 0.01	48.59 ± 0.18
Content of xylan (%)	21.55 ± 0.01	22.24 ± 0.06	21.67 ± 0.09	21.06 ± 0.17
Content of arabinose (%)	1.34 ± 0.06	1.41 ± 0.05	1.31 ± 0.07	1.27 ± 0.05
Content of lignin (%)	12.27 ± 0.21	11.86 ± 0.11	12.36 ± 0.03	12.94 ± 0.14
Solid recovery rate (%)	68.95 ± 0.12	68.74 ± 0.05	69.55 ± 0.57	71.01 ± 0.16
Recovery rate of glucan (%)	93.58 ± 0.11	94.90 ± 0.41	94.67 ± 0.75	94.86 ± 0.57
Recovery rate of xylan (%)	91.29 ± 0.22	93.89 ± 0.30	92.58 ± 0.36	91.84 ± 0.95
Recovery rate of lignin (%)	45.18 ± 0.68	43.55 ± 0.43	45.90 ± 0.25	49.07 ± 0.65
Lignin removal rate (%)	54.82 ± 0.68	56.45 ± 0.43	54.10 ± 0.25	50.93 ± 0.65
Lignin sulfonation degree (mmol/g)	1.12 ± 0.01	1.13 ± 0.02	1.12 ± 0.02	1.10 ± 0.01

Other pretreatment conditions are 7 wt.% KOH, 20 wt.% $(NH_4)_2SO_3$, 140°C, 2h.

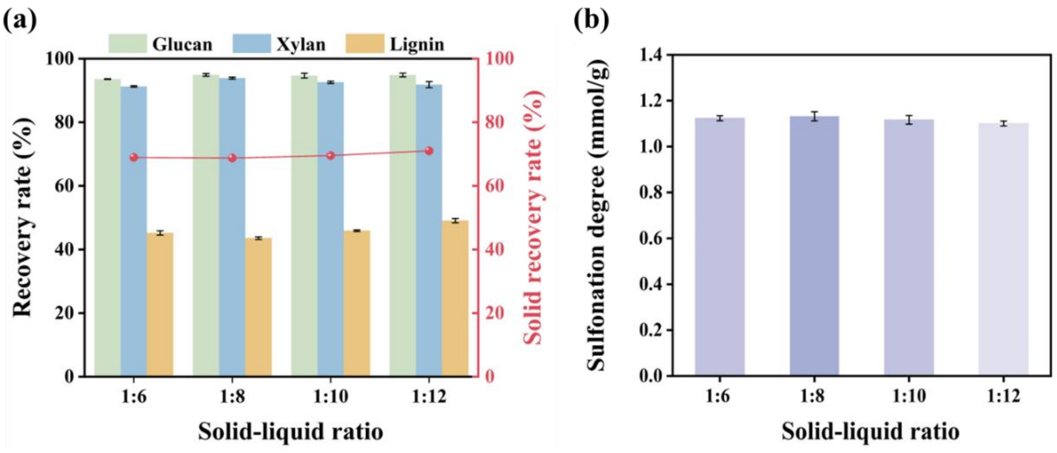

Figure S1. The influence of solid-liquid ratio on pretreatment effectiveness of *Miscanthus*. (a) Recovery rates of glucan, xylan and lignin; (b) sulfonation degree of the removed lignin.

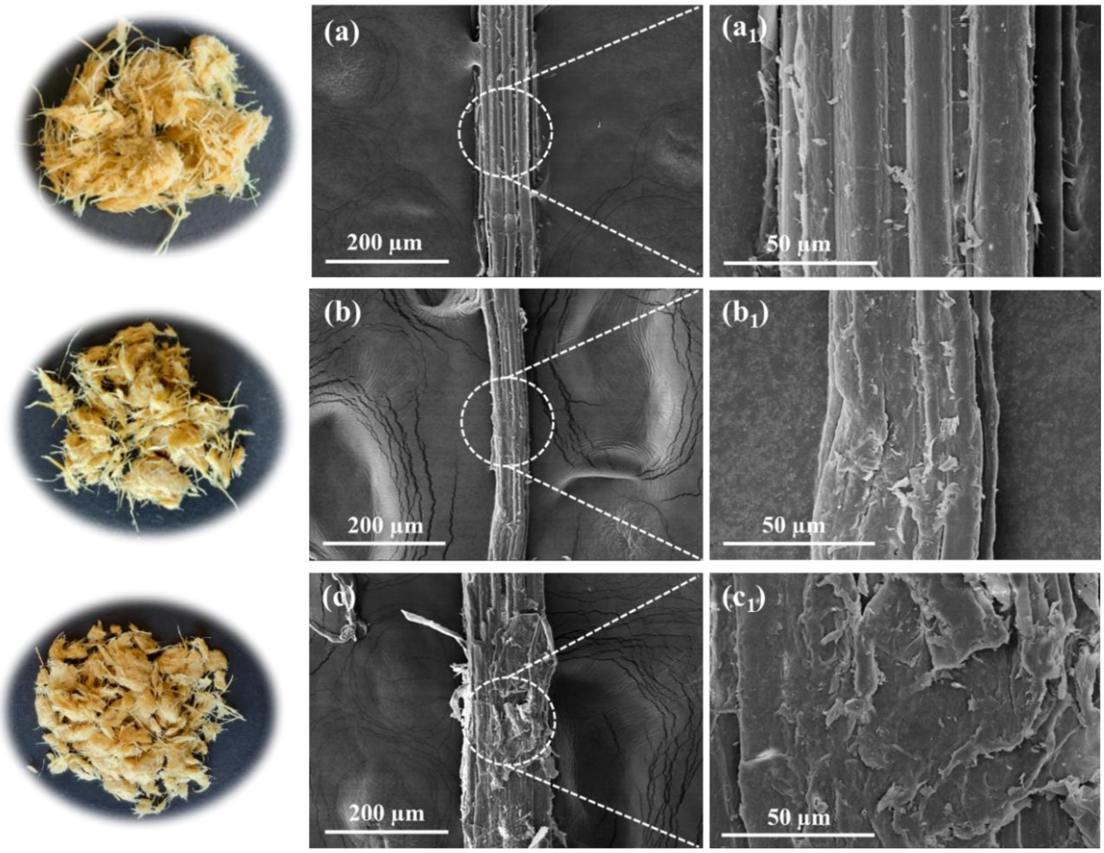

Figure S2. Appearance (left) and SEM images (right) of *Miscanthus* fibers after pretreatment with 15 wt.% $(NH_4)_2SO_3$ and PFI refining with different revolution numbers ((a) PFI revolution number of 1000 (b) PFI revolution number of 1500 (c) PFI revolution number of 2000).

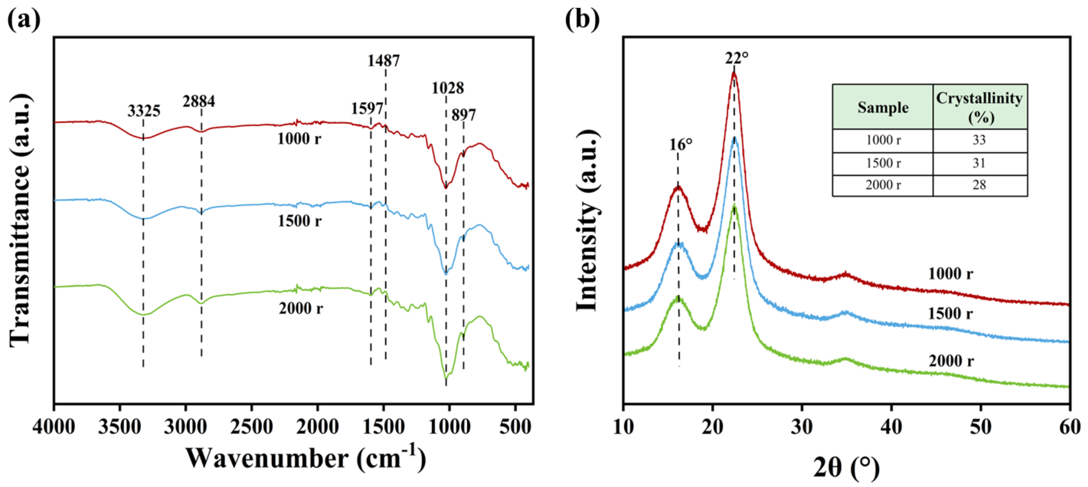

Figure S3. FTIR spectra (a) and XRD patterns (b) of *Miscanthus* fibers after pretreatment with 15 wt.% $(NH_4)_2SO_3$ and PFI refining with different revolution numbers.

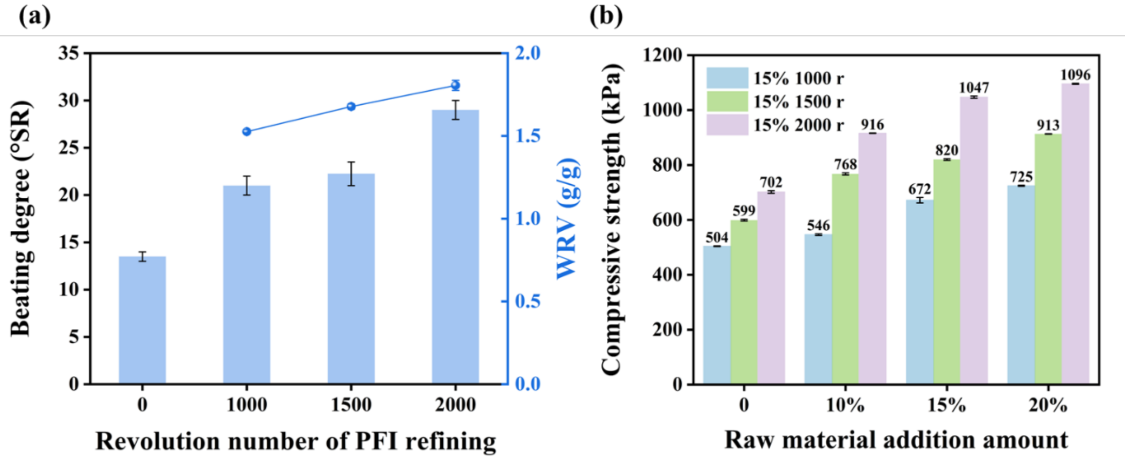

Figure S4. Beating degree and water retention value of *Miscanthus* fibers under different revolution numbers of PFI refining (a) and the compressive strength of *Miscanthus* seedling pots with the compressive strain of 80% (b). (The used pulp was the pretreated *Miscanthus* with 15 wt.% $(NH_4)_2SO_3$.

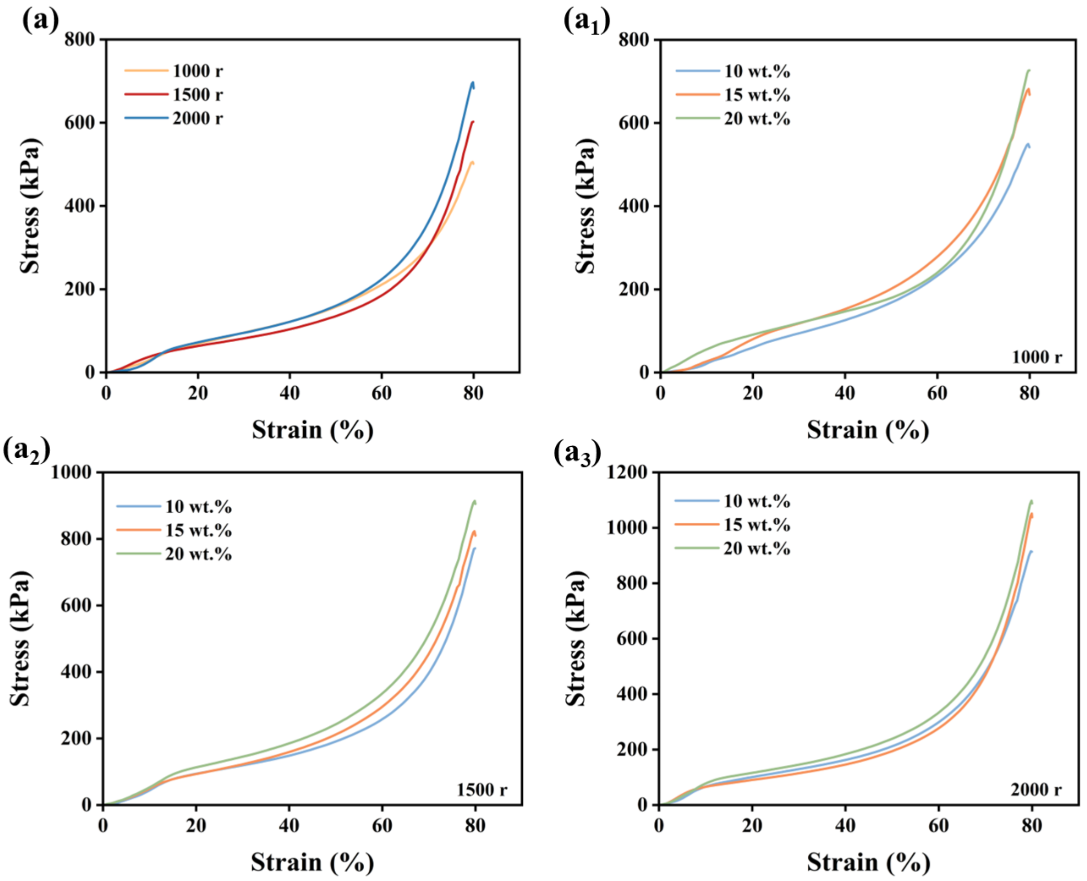

Figure S5. Stress-strain curves of *Miscanthus* seedling pots prepared using the pulp with 15 wt.% $(NH_4)_2SO_3$ and different PFI revolution numbers. Among them, (a_1), (a_2) and (a_3) are the stress-strain curves for the pot samples with different addition amounts of raw *Miscanthus* stalks when the revolution number of PFI refining are 1000, 1500, and 2000, respectively. The compressive strain is 80%.

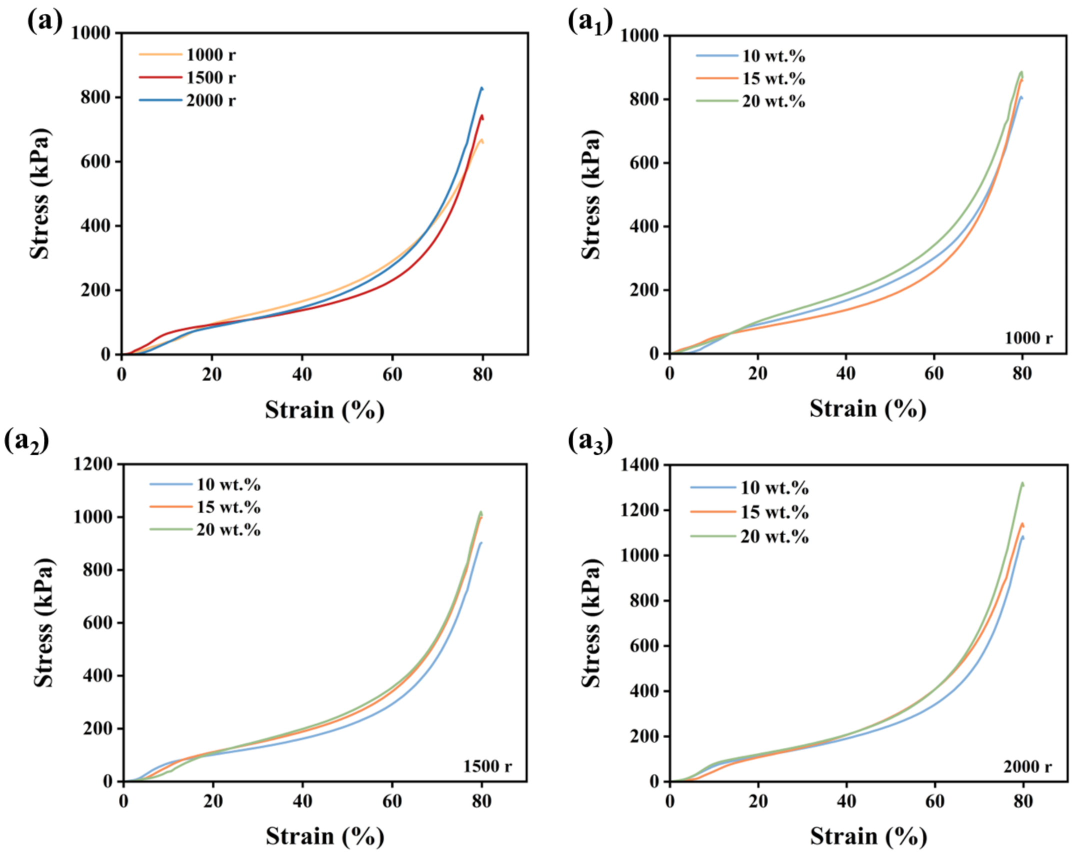

Figure S6. Stress-strain curves of *Miscanthus* seedling pots prepared using the pulp with 20 wt.% $(NH_4)_2SO_3$ and different PFI revolution numbers . Among them, (a_1), (a_2) and (a_3) are the stress-strain curves for the pot samples with different addition amounts of *Miscanthus* stalks when the revolution number of PFI refining are 1000, 1500, and 2000, respectively. The compressive strain is 80%.

Reference

1. Li, S.; Li, Z.; Zhang, Y.; Liu, C.; Yu, G.; Li, B.; Mu, X.; Peng, H. Preparation of concrete water reducer via fractionation and modification of lignin extracted from pine wood by formic acid. *ACS Sustain. Chem. Eng.* **2017**, 5, 4214–4222. doi:10.1021/acssuschemeng.7b00194.
